# Relationship Between the Uric Acid Level and CNS Relapse Risk in Patients with Newly Diagnosed Adult Diffuse Large B Cell Lymphoma

**DOI:** 10.3390/jcm15041642

**Published:** 2026-02-21

**Authors:** Abdullah Karakuş, Uğur Hatipoğlu, Vehbi Demircan, Orhan Ayyıldız

**Affiliations:** 1 Department of Hematology, Faculty of Medicine, Dicle University, 21280 Diyarbakır, Türkiye; abdullahkarakus10@gmail.com (A.K.);; 2 Department of Hematology & Apheresis Unit, Ankara Oncology Training and Research Hospital, University of Health Sciences, 06200 Ankara, Türkiye

**Keywords:** diffuse large B cell lymphoma, central nervous system involvement, International Prognostic Index, uric acid

## Abstract

**Introduction**: Central nervous system (CNS) involvement is reported to represent 5% of extranodal diffuse large B cell lymphoma (DLBCL) at diagnosis. To stratify patients’ risk of CNS involvement, the CNS-International Prognostic Index (CNS-IPI) score was developed. Serum uric acid level has not been incorporated into IPI or CNS-IPI score and its correlation with CNS-IPI risk groups and the CNS relapse has not been studied to date. Therefore, we aimed to retrospectively analyze the relationship between uric acid levels and CNS relapse risk in patients with newly diagnosed adult DLBCL. **Methods**: Ninety-four adult newly diagnosed DLBCL patients were retrospectively assessed via electronic records and patient files. Demographic data, IPI and CNS-IPI scores, hematologic parameters, serum lactate dehydrogenase, beta-2 microglobulin, serum uric acid and creatinine levels at diagnosis were recorded for analysis. Uric acid levels were compared between CNS-IPI low-intermediate and high groups and cumulative incidence of CNS relapses were analyzed between normal and elevated uric acid levels. **Results**: Uric acid levels were found to be significantly higher in CNS-IPI high-risk patients (*p* = 0.008). Elevated serum uric acid levels at diagnosis were significantly associated with high CNS-IPI risk in the logistic regression analysis (OR 1.34, 95% CI 1.05–1.78, *p* = 0.047). Uric acid also higher than 5.39 mg/dL showed a discriminatory ability in ROC analyses (AUC 0.633, 95% CI 0.495–0.771, *p* = 0.05). In the competing risks regression analysis, accounting for non-CNS-related death and progression as the competing events, CNS-IPI subgroups and uric acid levels were not significantly associated with the cumulative incidence of CNS relapse (SHR 0.81, 95% CI 0.12–5.59; Fine–Gray, *p* = 0.834), (SHR 0.55, 95% CI 0.09–3.47; Fine–Gray, *p* = 0.526), respectively. **Conclusions**: Even though uric acid levels are significantly higher and showed a discriminatory ability to detect the CNS-IPI high subgroup, elevated uric acid levels could not predict the CNS relapses.

## 1. Introduction

Diffuse large B cell lymphoma (DLBCL) is the most common lymphoma in western populations; accounting for nearly one-third of non-Hodgkin lymphomas. Nearly 150,000 cases of DLBCL are diagnosed worldwide each year [1,2]. DLBCL is more frequently seen in the seventh to eighth decades of life, and average age at diagnosis is 66 years; however, in some parts of the world, the median age at diagnosis is as low as 47.2 years [3,4]. DLBCL is clinically highly aggressive and biologically heterogenous; 40% of patients have extranodal involvement at time of diagnosis [5,6]. Central nervous system (CNS) involvement is reported to represent 5% of extranodal DLBCLs at diagnosis. CNS disease may also occur in relapsed/refractory settings, either isolated or synchronous with the systemic disease [7,8]. Primary CNS prophylaxis in DLBCL is still highly controversial in many aspects, like mode of prophylaxis (intrathecal versus intravenous) and patient selection. The prognosis of CNS relapse is extremely poor, with median survival ranging from 2 to 6 months [9,10].

To stratify patient’s risk of CNS involvement, CNS-International Prognostic Index (CNS-IPI) score was developed. Age, stage, number of extranodal sites, serum lactate dehydrogenase (LDH) level, performance status and kidney/adrenal involvement are the elements of CNS-IPI and each is assigned one point. Four points or greater is considered as high-risk of CNS relapse, where the risk of CNS disease development in 2 years is 10.2% [11]. Nevertheless, CNS-IPI lacks desired sensitivity; it predicted only half of CNS relapses in the study cohort. Anatomical location, cell of origin and double/triple hit status are not part of CNS-IPI but have been demonstrated to impact CNS relapse risk [10,11,12,13].

Tumor burden has been proposed as a prognostic variable in DLBCL and is correlated with LDH levels [14]. LDH is already incorporated into both the IPI and CNS-IPI score distinct from the stage itself [11,15]. In addition to LDH, uric acid—a degradation product of purine nucleotides—serves as a marker of nucleic acid metabolism within cells [16]. Values of 6.4 mg/dL or greater have been found to be an adverse prognostic finding for newly diagnosed, chemo or chemoimmunotherapy-treated patients [16]. Additionally, it was demonstrated that serum uric acid level is also an independent prognostic factor, beyond the established NCCN-IPI prognostic scoring system [17]. This was the first study that demonstrated that elevated serum uric acid is an unfavorable prognostic factor for overall and progression-free survival [17].

Current evidence regarding CNS-IPI driven prophylaxis with either intrathecal or systemic therapies is inconsistent. Therefore, there is an unmet need for tools that can predict the CNS relapses better than the CNS-IPI score. The heterogenous biology of DLBCL is considered as the major culprit for these uncertainties. This underscores the importance of exploring newer biomarkers to predict and reduce the CNS relapses [18,19].

Although serum uric acid level has not been incorporated into IPI or CNS-IPI scores, there is some evidence for its relevance to inferior outcomes, and its correlation with CNS-IPI risk groups has not been studied to date. Therefore, we aimed to analyze the relationships between uric acid levels, CNS-IPI risk stratification and CNS relapse rates.

## 2. Methods

### 2.1. Study Design and Subjects

**Chart 1:**
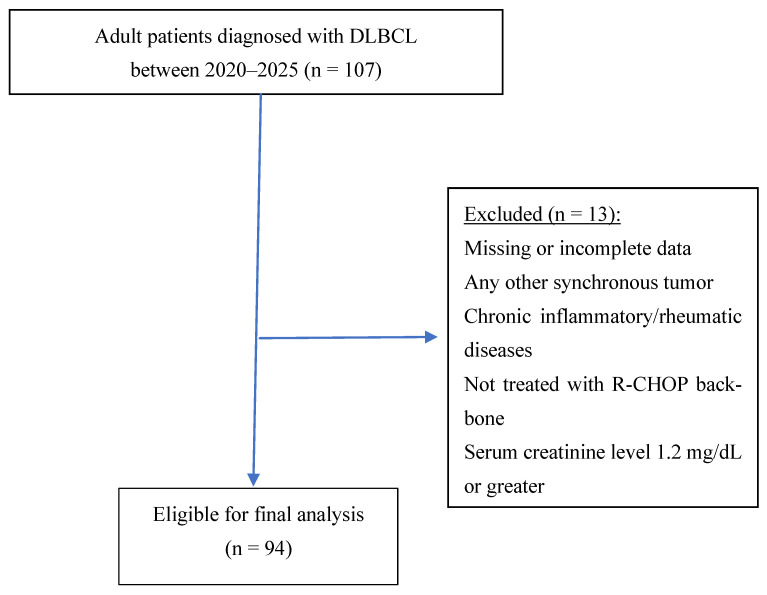
CONSORT diagram for study entry.

We conducted this single-center retrospective non-randomized observational study; a total of 107 adult newly diagnosed DLBCL patients in Dicle University, Diyarbakır-Türkiye, between 2020 and 2025 were assessed via electronic records and patient files. All patients included in the study were 18 and older. Exclusion criteria included patients with incomplete or missing key information, patients with other hematologic or non-hematologic malignancies, chronic inflammatory/rheumatic diseases and treatments other than rituximab plus cyclophosphamide, doxorubicin, vincristine and prednisone (R-CHOP) (Chart 1).

Demographic data, IPI and CNS-IPI scores, hematologic parameters, serum LDH, beta-2 microglobulin, serum uric acid and creatinine levels at diagnosis were recorded for analysis and those with a serum creatinine level of 1.2 mg/dL or greater were excluded as some biochemical parameters such as uric acid and beta-2 microglobulin are affected by renal functions [20]. Based on our center’s standard operation procedure, all biochemistry evaluations were completed before any therapeutic intervention (prophylaxis or chemoimmunotherapy). Our diagnostic workflow for suspected lymphoma remained unchanged between 2020 and 2025, and includes histopathologic examination, flow cytometry, complete blood count, chemistry and hepatitis panel. At final, a total of 94 patients were qualified as evaluable. Patients were divided into two groups according to the CNS-IPI: high (score of 4 or greater, n = 25) or CNS-IPI intermediate-low (scores between 0 and 3, n = 69).

### 2.2. Ethics Approval

Ethical approval was obtained from Diyarbakır Dicle University in accordance with the Declaration of Helsinki (approval number: 388/25), and written informed consent was obtained from all patients authorizing the use of their data for research purposes prior to the treatment.

### 2.3. Definitions

The Eastern Cooperative Oncology Group (ECOG) performance status is a standardized scale used to assess a patient’s functional status and ability to perform daily activities, ranging from 0 (fully active) to 4 (completely disabled) [21]. “Bulky” disease refers to the presence of a large tumor mass, and a cutoff of ≥7.5 cm is considered as bulky [22]. The term “B symptoms” refers to systemic manifestations used in lymphoma staging and prognostication: fever > 38 °C, drenching night sweats, and unexplained weight loss > 10% within 6 months [23]. The International Prognostic Index (IPI) was introduced to stratify patients with aggressive non-Hodgkin lymphomas based on five clinical factors: age > 60 years, stage III/IV disease, elevated LDH, ECOG ≥ 2, and >1 extranodal site. Risk groups are determined by summing these factors [24]. The CNS-IPI extends the original IPI by adding renal or adrenal involvement to identify patients at higher risk of central nervous system relapse. It stratifies DLBCL patients treated with R-CHOP into low-, intermediate-, and high-risk groups [11]. Progression-free survival (PFS) was estimated as the time between the first treatment and relapse, death, or last contact. Overall survival (OS) was defined as the time between the first treatment and death or last contact. In the cumulative incidence function (CIF) analyses of CNS relapse, progression rather than CNS relapse and death were regarded as the competitive risk factors.

### 2.4. Endpoints

The primary endpoint of this analysis was the correlation between plasma uric acid level and CNS-IPI scores and cumulative incidences of CNS relapse across two groups. Secondary endpoints were OS and PFS of the CNS-IPI subgroups.

### 2.5. Statistical Analysis

IBM SPSS Statistics version 26 and Stata SE v. 19.5 for Windows (Stata Corporation, College Station, TX, USA) were used to perform all the statistical analyses. The normality of distribution of continuous variables was assessed with the Kolmogorov–Smirnov test. Continuous variables were summarized as median and interquartile ranges, while categorical variables were expressed as numbers and percentages. For normally distributed variables, the independent samples *t*-test was applied; for non-normally distributed variables, the Mann–Whitney U test was used. Categorical variables were compared with the Chi-square test, or Fisher’s exact test when expected frequencies were <5. To identify the independent risk factors associated with high CNS-IPI, univariate logistic regression models were applied. Results were reported as odds ratios (ORs) with 95% confidence intervals (CIs). ROC curve analysis was performed to find the cut-off values of serum uric acid in high CNS-IPI patients. Reverse Kaplan–Meier analysis was used for median follow-up times. Survival function was assessed by the Kaplan–Meier method. Groups were compared using the log-rank test. CIF analyses of CNS relapse—progression rather than CNS relapse and death were regarded as the competitive risk factors—were performed for CNS-IPI high and low-intermediate groups. Patients who did not experience the failure event for the cumulative analyses were censored. Also, CIF analyses of CNS relapse were performed for all CNS-IPI parameters along with elevated uric acid levels. Continuous variables were dichotomized to avoid assuming a linear relationship with the outcome. Fine–Gray’s test was performed to determine the differences in the CIFs between groups. Confidence intervals (CI) were used as an estimate of variation within each group. A two-sided *p* value < 0.05 was considered statistically significant.

## 3. Results

### 3.1. Patient Characteristics

A total of 94 patients were included in the study: 52 patients (55.3%) were male and 42 patients (44.7%) were female, 51 patients (54.3%) were younger than 60 years, whereas 43 patients (45.7%) were aged 60 years or older. In total, 77.2% of cases presented with an ECOG performance status of 0–1, while 22.8% had an ECOG score ≥ 2. The advanced-stage population constituted more than half of cases, and 54.3% of patients were classified as stage IV, followed by 10.6% stage III, 22.3% stage II, 10.6% stage I, and 2.1% stage IE.

Evaluation of extranodal involvement showed that 38.3% of patients had no extranodal disease, 40.4% had involvement of a single extranodal site, and 21.3% had involvement of two or more extranodal sites. Bone marrow involvement was identified in only two patients (2.1%). Bulky disease was present in 21.3% of patients, and B symptoms were observed in 19.1%. Regarding histopathological subtypes, 54.3% of cases exhibited a non-germinal center B cell phenotype, 31.9% were of germinal center origin, and 13.8% were classified as unclassifiable. Laboratory evaluation revealed elevated lactate dehydrogenase (LDH) levels in 46.8% of patients, while 53.2% had LDH levels within the reference range of our center.

Risk stratification according to the CNS-International Prognostic Index (CNS-IPI) demonstrated that 46.8% of patients were categorized as low-risk, 26.6% as intermediate-risk, and 26.6% as high-risk. During follow-up, 12 patients died (12.8%), whereas CNS relapse was documented in five patients (5.3%). Relapse occurred in seven patients (7.4%) within follow-up while 10 patients (10.6%) exhibited primary refractory disease. The demographic and clinical data are presented in Table 1 and Table 2.

### 3.2. Comparison of Hematologic and Biochemical Parameters According to CNS-IPI Risk Groups

**Figure 1:**
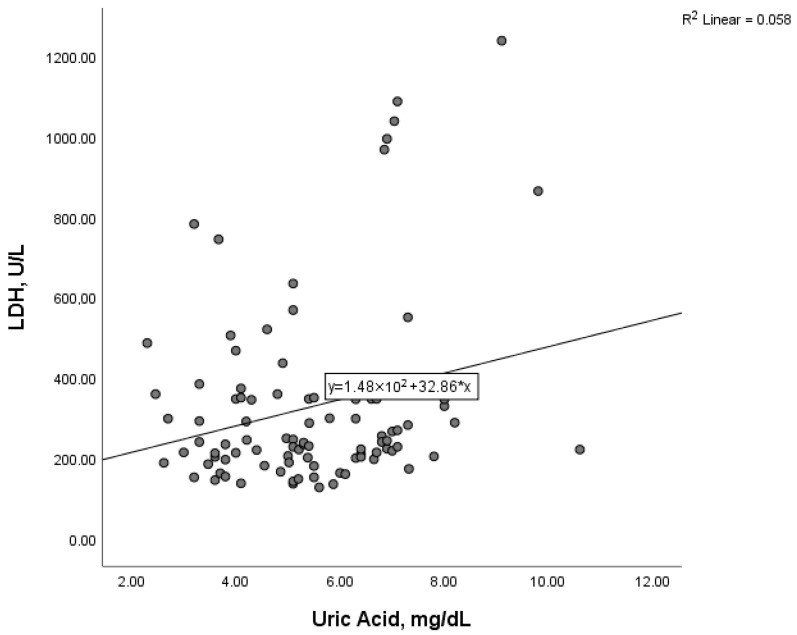
Pearson correlation analysis of LDH and uric acid (r: 0.241, *p* = 0.019).

All elements of the CNS-IPI score were consistently correlated with high CNS-IPI score. A total of 84% of the high CNS-IPI cohort had higher LDH; this ratio dropped to 33.3% in the low-intermediate cohort (*p* < 0.001). Patients with two or more extranodal disease were also greater in the CNS-IPI high cohort than intermediate-low (60% vs. 7.2%, *p* < 0.001), respectively. Age, ECOG of equal to or greater than two and advanced-stage disease were also statistically more prevalent in the CNS-IPI high cohort (*p* < 0.001 for all three variables). Neutrophil, platelet, and creatinine were not correlated with CNS-IPI risk scores (*p* = 0.92, *p* = 0.432 and *p* = 0.586). Mean hemoglobin level was nearly 2 gr/dL, much lower than the CNS-IPI low-intermediate and high-risk group, and this was statistically significant (*p* < 0.001). Mean uric acid level was also higher in the CNS-IPI high-risk group, and this was statistically significant but the difference was not pronounced as CNS-IPI elements or hemoglobin in the independent *t*-test (*p* = 0.046). Bulky disease (*p* = 0.036) and B symptoms (*p* = 0.002) were also significantly more prevalent among patients with high CNS-IPI scores. Similarly, beta-2 microglobulin levels were significantly higher among high-risk patients (*p* = 0.002). Pearson correlation analysis revealed a statistically significant correlation between serum LDH and uric acid levels (*p* = 0.019, Pearson’s r = 0.241) (Figure 1).

### 3.3. Logistic Regression Analyses for Factors Associated with High CNS-IPI Risk

Univariate logistic regression analyses demonstrated that poor performance status (ECOG ≥ 2) was strongly associated with high CNS-IPI risk (OR 22.0, 95% CI 6.5–74.9, *p* < 0.001). Moreover, involvement of two or more extranodal sites was independently associated with high CNS-IPI risk (OR 19.2, 95% CI 5.7–64.5, *p* < 0.001). Elevated LDH was significantly associated with high CNS-IPI classification (OR 10.5, 95% CI 3.22–34.2, *p* < 0.001). Advanced stage was also independently associated with high CNS-IPI risk (OR 20.7, 95% CI 2.6–162.1, *p* = 0.004). Age > 60 years was associated with a higher CNS-IPI score (OR 5.9, 95% CI 2.1–16.5, *p* = 0.001). Elevated serum uric acid levels were also significantly associated with high CNS-IPI risk in logistic regression analysis (OR 1.34, 95% CI 1.05–1.78, *p* = 0.047).

### 3.4. Receiver Operating Characteristic (ROC) Analysis

**Figure 2:**
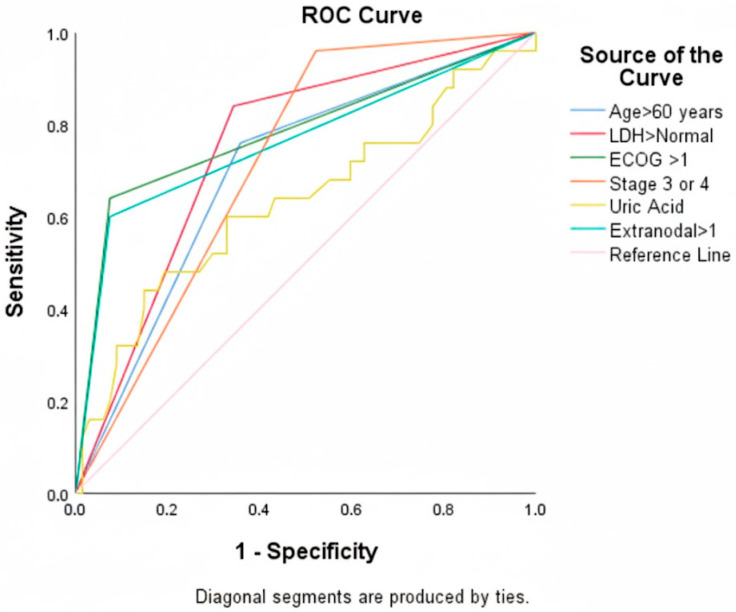
ROC curve analysis of discriminatory ability of age, LDH, ECOG > 1, advanced stage, >1 extranodal disease and uric acid.

Receiver operating characteristic (ROC) curve analyses were performed to assess the discriminative ability of selected variables for identifying high CNS-IPI risk. ECOG ≥ 2 demonstrated the highest diagnostic performance (AUC 0.783, 95% CI 0.662–0.904, *p* < 0.001), followed by ≥2 extranodal sites (AUC 0.763, 95% CI 0.638–0.888, *p* < 0.001), elevated LDH (AUC 0.748, 95% CI 0.639–0.858, *p* < 0.001), advanced stage (III–IV) (AUC 0.719, 95% CI 0.614–0.824, *p* = 0.001), and age > 60 years (AUC 0.701, 95% CI 0.582–0.820, *p* = 0.003). Serum uric acid also exhibited discriminative capacity (AUC 0.633, 95% CI 0.495–0.771, *p* = 0.05). Uric acid levels greater than 5.39 mg/dL indicated high CNS-IPI risk with 64% sensitivity and 57% specificity in ROC analysis (Figure 2).

### 3.5. Survival Analysis

**Figure 3:**
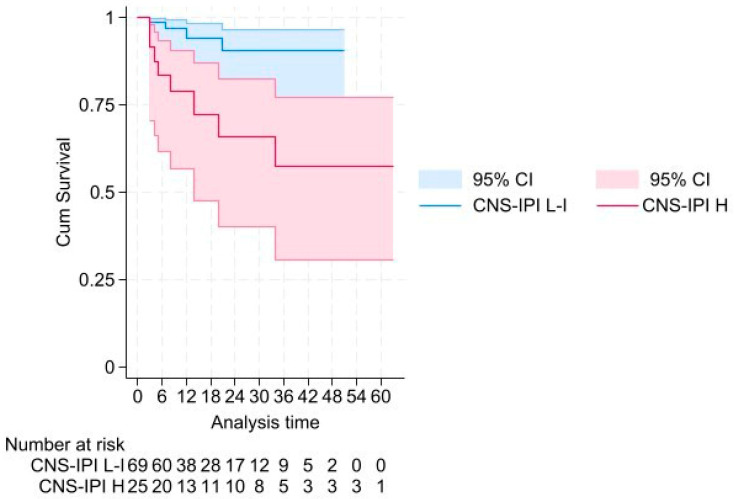
Kaplan–Meier plot for OS (log-rank *p* = 0.003).

**Figure 4:**
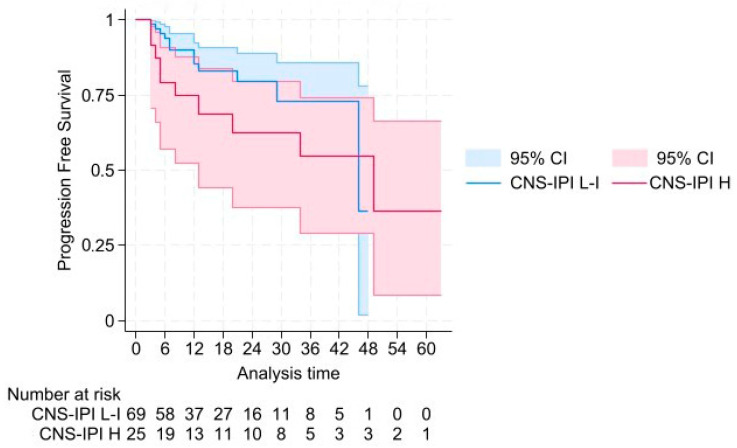
Kaplan–Meier plot for PFS (log-rank *p* = 0.168).

Median follow-up was 27 months (1–63) in the CNS-IPI high group and 15 months (1–51) for the CNS-IPI low-intermediate group. Median OS was not reached both in CNS-IPI low-intermediate and high groups, while median PFS was 46 and 49 months, respectively. Kaplan–Meier analyses demonstrated a significant difference in OS according to CNS-IPI risk status (log-rank *p* = 0.003) (Figure 3), whereas no significant difference in PFS was observed across the risk groups (log-rank *p* = 0.168) (Figure 4).

### 3.6. Cumulative Incidence of CNS Relapse

**Figure 5:**
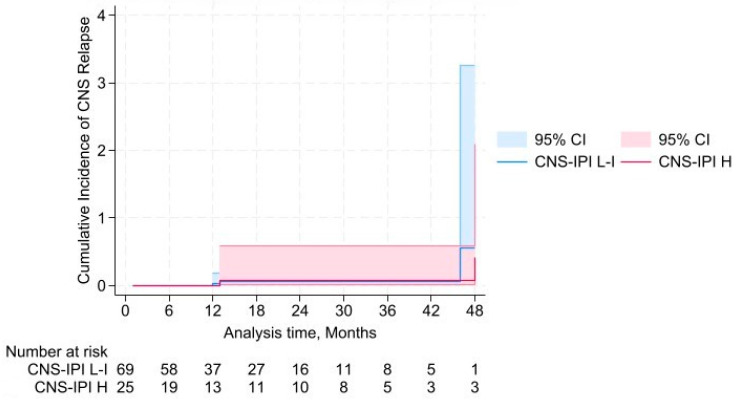
Cumulative incidence of CNS relapse across groups. Progression and death were the competitive risk factors.

Five patients had CNS relapse during the follow up time. Two female patients, 62 and 78 years old, had in the CNS-IPI high group, where relapses occurred at the 13th and 48th months, respectively; two female patients, 58 and 77 years old, had in the CNS-IPI intermediate group, where relapses occurred at the 12th and 13th months, respectively; and only one male patient, 67 years old, had in the CNS-IPI low group, and relapse occurred in this patient at the 46th month. In the competing risks regression analysis for the CNS-IPI low-intermediate and high groups accounting for non-CNS-related death and progression rather than CNS relapse as competing events, CNS-IPI was not significantly associated with the cumulative incidence of CNS relapse (SHR 0.81, 95% CI 0.12–5.59; Fine–Gray, *p* = 0.834) (Figure 5).

### 3.7. Comparisons of Cumulative Incidences of CNS Relapse with All CNS-IPI Parameters and Uric Acid Levels

**Figure 6:**
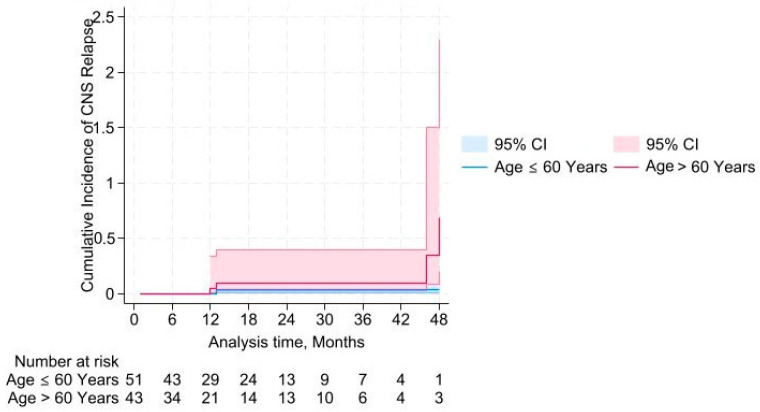
Cumulative incidence of CNS relapse according to age. Progression and death were the competitive risk factors.

**Figure 7:**
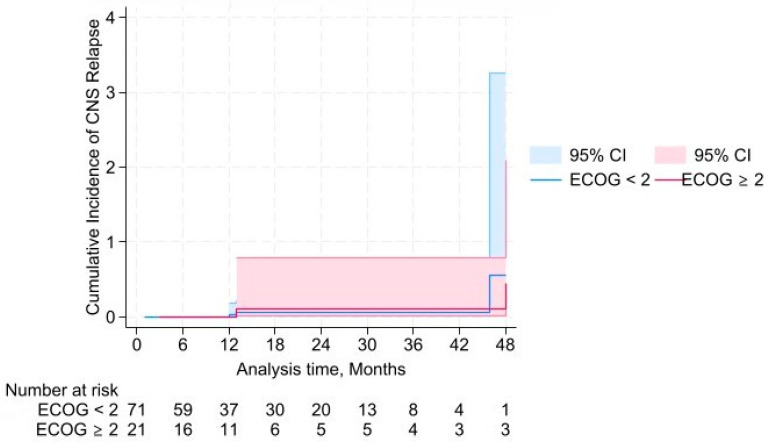
Cumulative incidence of CNS relapse according to the ECOG. Progression and death were the competitive risk factors.

**Figure 8:**
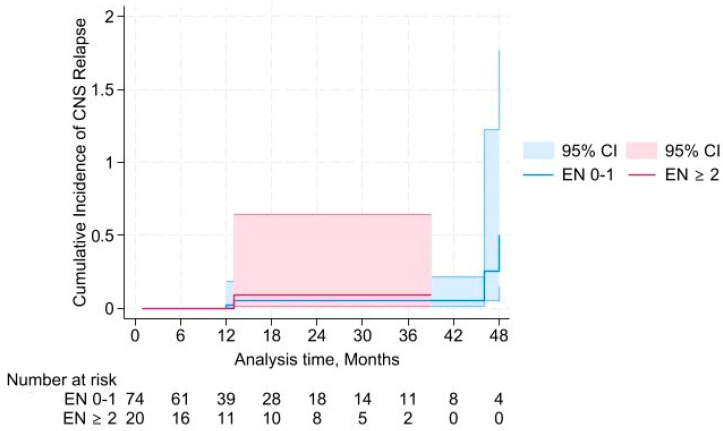
Cumulative incidence of CNS relapse according to the extranodal involvement. Progression and death were the competitive risk factors.

**Figure 9:**
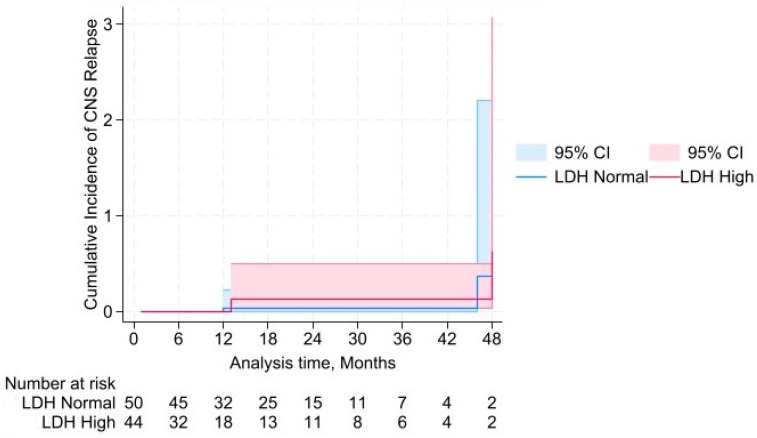
Cumulative incidence of CNS relapse according to the elevated LDH. Progression and death were the competitive risk factors.

**Figure 10:**
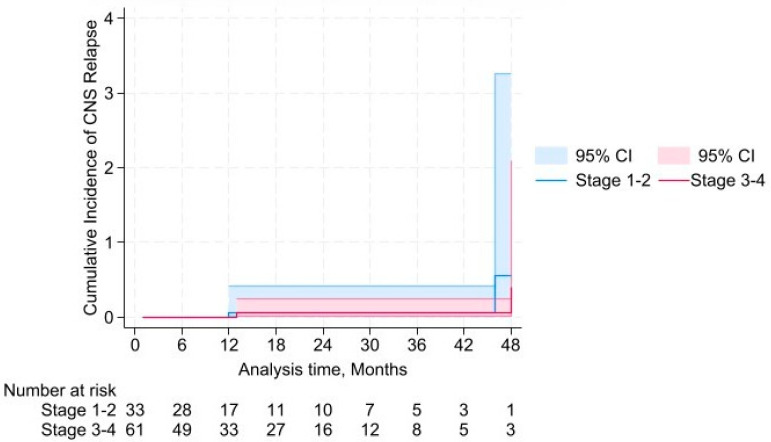
Cumulative incidence of CNS relapse according to the stages. Progression and death were the competitive risk factors.

**Figure 11:**
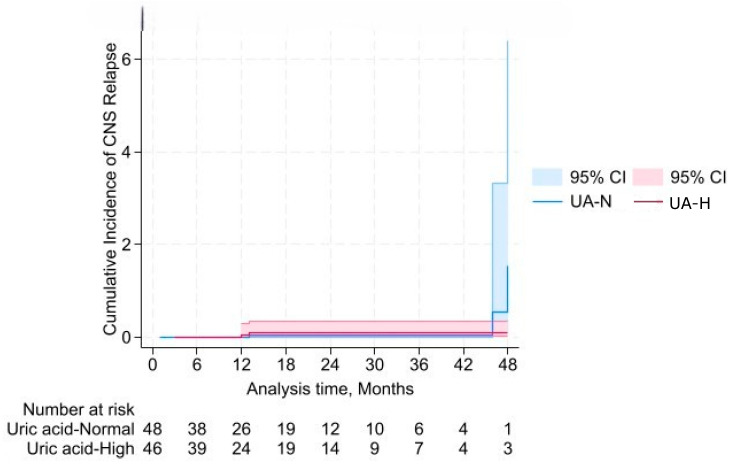
Cumulative incidence of CNS relapse according to the uric acid levels. Progression and death were the competitive risk factors.

Age > 60 years (SHR 3.68, 95% CI 0.43–31.01; Fine–Gray, *p* = 0.230); ECOG ≥ 2 (SHR 0.79, 95% CI 0.10–6.06; Fine–Gray, *p* = 0.824); extranodal involvement ≥ 2 (SHR 1.34, 95% CI 0.15–11.3; Fine–Gray, *p* = 0.786); elevated LDH (SHR 1.75, 95% CI 0.29–10.18; Fine–Gray, *p* = 0.534); stage 3–4 (SHR 0.62, 95% CI 0.11–3.31; Fine–Gray, *p* = 0.578); and elevated uric acid (SHR 0.55, 95% CI 0.09–3.47; Fine–Gray, *p* = 0.526) were not significantly associated with the cumulative incidence of CNS relapse (Figure 6, Figure 7, Figure 8, Figure 9, Figure 10 and Figure 11).

## 4. Discussion

This is the first study that has investigated whether uric acid levels predict CNS relapses, and the main findings of our study were; (i) uric acid levels was found to be significantly higher in CNS-IPI high-risk patients, (ii) elevated serum uric acid levels were significantly associated with high CNS-IPI risk, (iii) uric acid levels greater than 5.39 mg/dL indicated high CNS-IPI risk with 64% sensitivity and 57% specificity, and (iv) CNS-IPI parameters and uric acid levels were not significantly associated with the cumulative incidence of CNS relapse.

As mentioned earlier, tumor burden is an important prognostic measure [14]. LDH and number of extranodal sites are robust markers of tumor burden and therefore they are closely related to outcomes [25]. Kim et al. have demonstrated that an LDH level exceeding x3 ULN is a strong predictor of CNS relapse [25]. Disease sites are constantly being subject to debate regarding CNS dissemination. Testicular involvement was considered a high-risk stigmata for spreading to the CNS, although studies from the rituximab era strongly questioned this concept [26,27,28]. In addition to the testis, bone marrow involvement is an area of mixed evidence. Despite being dismissed by CNS-IPI creators, a Swedish group found bone marrow disease to be an independent risk factor for CNS involvement [27]. Breast involvement is also not included in the CNS-IPI score but a recent study demonstrated its high-risk [28]. Kidney and/or adrenal involvement are the only factors that retained their predictive value in both univariate and multivariate analysis of the test and validation cohorts of the study [11]. Therefore, kidney and adrenal are the only extranodal sites featuring in the CNS-IPI model [11]. Disease stage is a direct reflection of the burden itself and is included in the CNS-IPI score [11]. Based on its tumor burden surrogate domains, CNS-IPI score captures elements of overall tumor bulk.

Uric acid also was proposed as a prognostic marker for newly diagnosed DLBCL [16,17]. Prochaska et al. set the threshold as 6.8 mg/dL for poorer outcomes, while Tian et al. set this value slightly lower [16]. The former study has demonstrated that serum uric acid level at diagnosis is associated with inferior prognosis independently of NCCN-IPI score [17]. Ywei et al. incorporated serum uric acid level into the traditional IPI score, and the new tool performed better in terms of OS and PFS. The proposed threshold for serum uric acid was 6.4 mg/dL in this research [16]. A retrospective analysis in Thailand clearly indicated that serum uric acid level is well correlated with serum LDH, a reliable marker of tumor burden [29]. Furthermore, several other studies have consistently shown that serum uric acid level is a good reflection of overall tumor burden and prognosis for various types of cancer [30,31,32]. In lung cancer but not lymphoma, higher levels of serum uric acid was associated with asymptomatic CNS metastasis [33]. In our study, uric acid levels were found to be significantly higher in CNS-IPI high-risk patients and elevated serum uric acid levels were significantly associated with high CNS-IPI.

Despite these intense efforts, predicting CNS relapse in DLBCL is still challenging. CNS-IPI score is composed of clinical variables, where no genetic/immunological factors are represented [11]. Furthermore, CNS-IPI score was found to be insufficient in some circumstances. As an example, myeloid differentiation primary response gene 88 mutation L265P (*MYD88*^L265P^) and/or cluster differentiation 79B gene mutation Y196 (*CD79B*^Y196^) (*MYD88*/*CD79B*) are two strong markers that would be strongly suggestive of CNS relapse, even for low-intermediate CNS-IPI risk [34]. Moreover, a distinct cytokine profile was proposed as a surrogate for CNS dissemination [35]. Newer approaches to determine CNS relapse risk will continue being subject to further research, and, as we reported with uric acid levels in this paper, CNS-IPI score is not accurate enough.

Certain limitations of the present study should be considered. First, it used a single-center retrospective non-randomized study design, with a relatively small number of patients and CNS events, and the follow-ups of the our whole cohort were short.

In conclusion, our study identifies serum uric acid as a significant biochemical indicator for high-risk CNS-IPI scores in patients with DLBCL. The overall clinical impact of this finding lies in providing an accessible and cost-effective tool for refined risk stratification, potentially aiding clinicians in identifying high-risk patients who may require more vigilant monitoring or intensive prophylactic strategies.

Even though uric acid levels showed a strong discriminatory ability to detect the CNS-IPI high-risk subgroup, they were not independent predictors of actual CNS relapses in this cohort. This suggests that while elevated uric acid is a robust surrogate for high tumor burden and aggressive biology—both of which are captured by the CNS-IPI—further studies are needed to determine if it reflects a direct biological mechanism for CNS infiltration. As the first paper to demonstrate this specific association, our analysis serves as a foundational step. Integrating uric acid levels into existing prognostic models could enhance their accuracy, and we believe these results will catalyze future prospective trials aimed at validating metabolic biomarkers to improve the prevention and management of CNS involvement in DLBCL.

## Figures and Tables

**Table 1:** Demographics and laboratory values of the patients.

Variables	CNS-IPI High-Risk (n = 25)	CNS-IPI Low or Intermediate Risk (n = 69)	*p* Value
Age, years	64.0 (60.0–70.5)	58.0 (43.5–66.0)	0.002 ^a^
Gender, n (%) Male Female	11 (44) 14 (56)	41 (59.4) 28 (40.6)	0.184 ^b^
Leucocyte count, /uL	10,000 (6800–10,725)	8320 (4350–6000)	0.152 ^a^
Lymphocyte count, /uL	1400 (895–2200)	1640 (1315–2145)	0.236 ^a^
Neutrophil count, /uL	5670 (3685–8790)	5330 (3740–7050)	0.928 ^c^
Monocyte count, /uL	700 (525–1020)	520 (395–800)	0.029 ^a^
Hemoglobin, g/dL	11.30 (9.60–12.95)	13.60 (12.05–15.00)	<0.001 ^c^
Platelet, /uL	310,000 (227,500–401,000)	277,000 (207,500–333,500)	0.432 ^c^
Beta-2 microglobulin, mg/L	3.62 (2.21–5.30)	2.16 (1.64–3.00)	0.002 ^a^
LDH, U/L	467 (346–804)	221 (186–289)	<0.001 ^a^
Creatinine, mg/dL	0.72 (0.62–0.86)	0.76 (0.65–0.88)	0.560 ^c^
Uric acid, mg/dL	6.30 (4.30–7.10)	5.10 (4.05–6.40)	0.008 ^c^

Mann–Whitney U ^a^, Pearson chi-square ^b^, independent samples *t*-test ^c^; continuous variables were expressed as median-interquartile ranges; categorical variables were expressed as numbers and percentages. Abbreviations: LDH: Lactate Dehydrogenase.

**Table 2:** Clinical characteristics of the patients.

Variables	CNS-IPI High-Risk (n = 25)	CNS-IPI Low or Intermediate Risk (n = 69)	*p* Value
ECOG, n (%) 0–1 2–3	9 (36) 16 (64)	62 (89.8) 7 (10.2)	<0.001 ^a^
Bulky disease, n (%)	9 (36)	11 (15.9)	0.036 ^a^
B symptoms, n (%)	10 (40)	8 (11.6)	0.006 ^b^
Cell of origin, n (%)			
Germinal	2 (8)	28 (40.6)	
Non-Germinal	17 (68)	34 (49.3)	0.007 ^a^
Undetermined	6 (24)	7 (10.1)	
Extranodal Involvement, n (%)			
None	2 (8)	34 (49.3)	
Single	8 (32)	30 (43.5)	<0.001 ^a^
Two or more	15 (60)	5 (7.2)	
Stage, n (%)			
1–2	1 (4)	32 (46.3)	<0.001 ^a^
3–4	24 (96)	37 (53.7)	

Pearson chi-square ^a^, Fisher exact test ^b^. Variables were expressed as numbers and percentages. Abbreviations: ECOG: Eastern Clinical Oncology Group.

## Data Availability

The original contributions presented in this study are included in the article. Further inquiries can be directed to the corresponding author.
